# MSTT: A Multi-Spatio-Temporal Graph Attention Model for Pedestrian Trajectory Prediction

**DOI:** 10.3390/s25154850

**Published:** 2025-08-07

**Authors:** Qingrui Zhang, Xuxiu Zhang, Zilang Ye, Jing Mi

**Affiliations:** 1 School of Automation & Electrical Engineering, Dalian Jiaotong University, Dalian 116028, China; qr_zhang2025@163.com; 2 School of Mechanical Engineering, Dalian Jiaotong University, Dalian 116028, China; 19820506327@163.com; 3 Department of Mathematics, University of Padova, 35100 Padova, Italy; jing.mi@phd.unipd.it

**Keywords:** pedestrian trajectory prediction, attentional mechanism, graph structure learning

## Abstract

Accurate prediction of pedestrian movements is vital for autonomous driving, smart transportation, and human–computer interactions. To effectively anticipate pedestrian behavior, it is crucial to consider the potential spatio-temporal interactions among individuals. Traditional modeling approaches often depend on absolute position encoding to discern the positional relationships between pedestrians. Unfortunately, this method overlooks relative spatio-temporal relationships and fails to simulate ongoing interactions adequately. To overcome this challenge, we present a relative spatio-temporal encoding (RSTE) strategy that proficiently captures and analyzes this essential information. Furthermore, we design a multi-spatio-temporal graph (MSTG) modeling technique aimed at modeling and characterizing spatio-temporal interaction data across several individuals over time and space, with the goal of representing the movement patterns of pedestrians accurately. Additionally, an attention-based MSTT model has been developed, which utilizes an end-to-end approach for learning the structure of the MSTG. The findings indicate that an understanding of an individual’s preceding trajectory is crucial for forecasting the subsequent movements of other individuals. Evaluations using two challenging datasets reveal that the MSTT model markedly outperforms traditional trajectory-based modeling methods in predictive performance.

## 1. Introduction

With the rapid advancement of autonomous driving technology, accurately predicting pedestrian trajectories has become a critical task for ensuring the safety of both vehicles and pedestrians [1,2,3]. In the complex and unpredictable urban environment, the variability and unpredictability of pedestrian interactions pose significant challenges to the perception and decision-making systems of autonomous vehicles. Precise trajectory prediction has been shown to reduce collision rates of autonomous vehicles in high-density urban traffic by 10% to 20% [4] in closed-loop testing.

Predicting the future movements of pedestrians in complex environments presents a significant challenge due to the high subjectivity and randomness of human interactions. Empirical methods explicitly model interactions to predict crowd motion, e.g., a rule-based model [5], a force-based model [6] and an energy-based model [7]. However, due to the inability to precisely fit the observed data in dynamic and changing environments, the model exhibits poor generalization, resulting in a decrease in predictive accuracy during closed-loop testing. In contrast, various methods based on deep neural networks have been proposed for pedestrian interaction modeling by employing social pooling layers [8,9,10,11], graph neural networks (GNNs) [12,13,14,15], and attention mechanisms [16]. While they demonstrate strong expressive power in open-loop testing and exhibit some generalization ability in closed-loop testing, the black-box nature of neural networks limits their interpretability. Exploring the trade-off between model explainability and prediction capability remains a challenging task.

**Figure 1:**
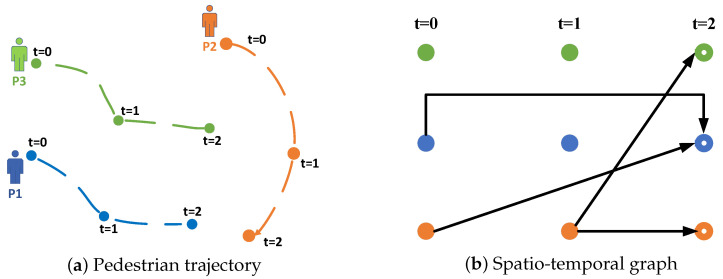
(**a**) depicts the trajectories recorded for several agents during the time from t = 0 to t = 2. Most conventional models presume a static structure; (**b**) illustrates a multi-spatio-temporal graph framework employed to forecast the trajectories of many actors at time t.

The integration of graph neural networks with Transformers has been rigorously studied in this field [17,18]. While this convergence has led to performance improvements, further research is needed to better understand its connection to pedestrian behavior. Figure 1 illustrates that earlier approaches [15] typically assumed that pedestrian interactions were based on geographical correlations from prior encounters. However, the current focus on spatio-temporal interactions at specific time points—either past or future—renders these methods insufficient for accurately capturing pedestrian interactions. Moreover, most models rely on Transformer-based absolute position encoding to integrate pedestrian data, which limits adaptability, as these models cannot easily adjust their network parameters. This issue is exacerbated by the frequent appearance and disappearance of pedestrians. Some studies [19,20] have adopted an evolutionary strategy for multi-agent trajectory prediction to improve adaptability. However, these approaches are often formulated at discrete time intervals, neglecting the continuous nature of pedestrian interactions and failing to fully represent the dynamic behavior of pedestrians.

**Figure 2:**
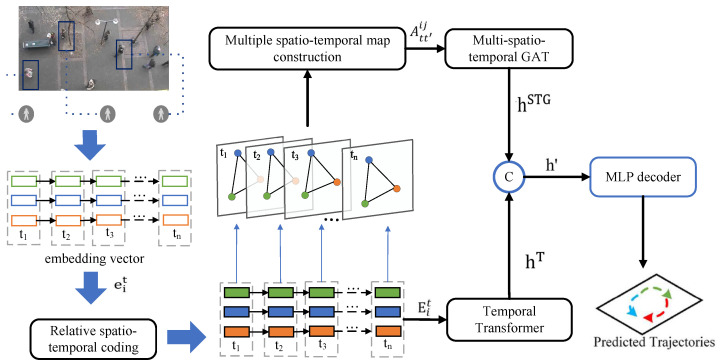
The MSTT model consists of two principal components: the encoder and the decoder. The encoder integrates modules for relative spatio-temporal encoding and multi-spatio-temporal fusion. The module for relative spatio-temporal encoding takes as input the position of pedestrians, their identification, and the frame number. The resultant encoded output is processed by a temporal Transformer to encapsulate pedestrian temporal dynamics and is subsequently introduced into the multi-spatio-temporal fusion module to model interactions and derive the adjacency matrix. The multi-spatio-temporal GAT along with the temporal Transformer are then employed to train and generate interaction state information. The decoder leverages a multi-layer perceptron (MLP) to interpret the interaction data, yielding the corresponding real-world 2D coordinates.

We introduce a novel trajectory prediction model, the Multiple-Relative Spatio-Temporal Graph Transformer (MSTT), which is designed to model the complex interactions among pedestrians in the spatio-temporal domain. This model integrates relative spatio-temporal encoding with advanced spatio-temporal modeling techniques to predict pedestrian trajectories accurately, as shown in Figure 2. Unlike traditional Transformer models that rely on absolute position encoding [21], the MSTT employs a relative spatio-temporal encoding strategy, which captures the dynamic spatio-temporal dependencies across various pedestrian nodes during encoding. This approach enables the model to better understand intricate pedestrian interactions, including behaviors that may be hidden or absent, thereby enhancing the model’s flexibility and adaptability. Ultimately, the MSTT introduces a multi-graph fusion technique that captures continuous spatio-temporal interdependence among pedestrians, ensuring that all interactions are modeled simultaneously, rather than treating spatial and temporal relationships separately.

The proposed methodology was evaluated on several challenging datasets, including ETH [22], UCY [23], SDD [24], and the SportVU NBA sports dataset. The experimental results demonstrated that our model significantly improved the accuracy of pedestrian trajectory prediction. Ablation studies of various model components further substantiated its effectiveness. The primary aim of this study was to model the dynamic interactions and temporal dependencies that enhance the accuracy of pedestrian trajectory prediction techniques.

The main contributions of this study are summarized as follows:

We introduce a comprehensive model for predicting pedestrian trajectories, known as the MSTT, which harnesses the dynamic interactions among pedestrians within varied spatio-temporal frameworks to project their forthcoming movements.

We propose a relative spatio-temporal coding methodology that employs the encoding of periodic traits to encapsulate the cyclic nature of spatio-temporal interactions by illustrating the relative distinctions between temporal and spatial dimensions. This enables the model to adeptly manage diverse spatio-temporal intervals and effectively reduce bias towards specific nodes.

We have developed an advanced spatio-temporal graph modeling methodology that evaluates pedestrian interaction links across varied spatio-temporal intervals. This is achieved through the superimposition of spatial graphs from different time points, application of dynamic thresholding using multimodal data, and the ultimate creation of numerous spatio-temporal graphs. These graphs are produced by filtering pivotal occurrences predicated on the intensity of interactions.

Comprehensive experiments conducted on publicly accessible pedestrian trajectory datasets substantiate that our proposed algorithm surpasses the performance of several baseline methodologies, including state-of-the-art algorithms.

## 2. Related Work

### 2.1. Multi-Intelligent Body Trajectory Prediction

The relationship between autonomous cars and pedestrians has attracted a lot of interest lately, especially when it comes to mixed urban traffic situations [21]. To improve road safety and maximize the operational effectiveness of autonomous driving systems in these intricate, dynamic situations, it is critical to be able to anticipate pedestrian behaviour and how they will interact with vehicles.To address this challenge, Alahi et al. [15] utilized long short-term memory (LSTM) networks to deduce latent states, which were then disseminated to adjacent pedestrians. PITF [25] incorporates pedestrian behavior and interaction modules, effectively incorporating visual information into its feature set. STGAT [26] is the inaugural model that integrates graph attention networks (GATs) with LSTM to model pedestrian movements. PECNet [27] employs conditional variational autoencoders (CVAEs) [28] to deduce trajectory endpoints, enhancing the precision and dependability of trajectory forecasts.

Moreover, with the rapid advancement of autonomous driving technology, interactions between pedestrians and autonomous vehicles have become a central focus of research. For example, the IAMPDM method [29] combines deep learning with decision-making frameworks to optimize autonomous vehicle behavior through pedestrian intent recognition. In parallel, cooperative decision-making models [30] leverage cooperative control strategies and model predictive control (MPC) to predict pedestrian motion and adjust vehicle behavior accordingly, thereby improving adaptability and enhancing traffic safety. Furthermore, due to the frequent interactions between vehicles and pedestrians, recent work has focused on optimizing model size to accelerate inference. For instance, DERGCN [19] employs evolving graph convolutional networks to reduce model parameters, thus improving the inference speed. DSDrive [31] introduces a waypoint-based dual-head coordination module to synchronize data structures, optimization objectives, and training procedures.

### 2.2. Dynamic Graph Neural Networks

In recent years, substantial progress has been achieved in the study of dynamic graph neural networks (DGNNs) and Transformers [32,33]. Dynamic graphs are characterized by the emergence, disappearance, or reconnection of nodes and edges at various intervals of time. DGNNs represent a specialized neural network architecture crafted for dynamic networks; they are adept at capturing the temporal evolution of nodes and edges while encoding the attributes of neighboring nodes throughout this process [34,35,36,37]. Currently, DGNNs conventionally integrate graph neural networks (GNNs) to encapsulate the structural attributes of the graph, alongside Transformers or temporal neural networks to discern temporal patterns. DGNNs can be categorized into discrete and continuous types depending on how they manage graph structures. Discrete approaches employ static GNNs at each time interval to extract information from the dynamic graph, whereas continuous methods integrate the temporal dimension directly into the representation learning of dynamic graphs, thereby facilitating the dynamic learning of spatio-temporal interactions.

### 2.3. Transformer-Based Trajectory Prediction

Given the proven success of the Transformer architecture in natural language processing (NLP) and computer vision (CV), an increasing number of studies have adopted this framework for trajectory prediction tasks. For example, Yu et al. [16] utilized a spatial Transformer to model spatial relationships among pedestrians, along with a temporal Transformer to capture sequential dependencies. Similarly, Zhou [9] calculated similarity coefficients between node pairs, applied supervised adjacency matrices, and employed a group-aware spatio-temporal Transformer for model training.

In this research context, Transformer-based models such as the knowledge-aware graph Transformer [38], MGFormer [39], and STGSTN [40] effectively handle spatio-temporal interactions via attention mechanisms, demonstrating their potential in complex dynamic environments. While these graph-based Transformers are proficient in modeling long-range dependencies and complex inter-agent relationships, they fall short in capturing the social and spatio-temporal nuances of pedestrian interactions. To address this limitation, we propose the MSTT model, which integrates pedestrian social information and spatio-temporal graph structures with relative positional encoding. This approach significantly enhances trajectory prediction accuracy.

## 3. Approach

### 3.1. Problem Formulation

Consider a scenario where there are *N* pedestrians $p_{i}$, with i∈{1,2,…,N}. At a specific time *t*, the position of pedestrian $p_{i}$ is $l_{i}^{t}=\left(x_{i}^{t},y_{i}^{t}\right)$. The objective is to predict the future trajectory $l_{i}^{t}$ of the pedestrian at future times $t\in \{t_{n+1},t_{n+2},\ldots ,t_{pred}\}$ based on the observed positions $l_{i}^{t}$ during the time interval $t=1,\ldots ,t_{n}$ and the interactions between pedestrians.

### 3.2. Trajectory Coding for a Pedestrian

The proposed MSTT model first computes a set of motion-related features for each pedestrian $p_{i}$ at time *t*, defined as

(1) $$f_{i}^{t}=\left[l_{i}^{t},\Delta x_{i}^{t},\Delta y_{i}^{t},v_{i}^{t},\alpha_{i}^{t},\theta_{i}^{t}\right]$$

where $l_{i}^{t}$ signifies the spatial coordinates of pedestrian $p_{i}$ at time *t*, whereas $\Delta x_{i}^{t}$ and $\Delta y_{i}^{t}$ indicate the lateral and longitudinal displacements, respectively. $v_{i}^{t}$ denotes the instantaneous velocity; $\alpha_{i}^{t}$ marks the acceleration, and $\theta_{i}^{t}$ represents the current bearing. The encoder handles the MLP input subsequent to its conversion into a fixed-length feature vector, $e_{i}^{t}$, for further processing by downstream models. This transformation is described as follows:

(2) $$e_{i}^{t}=\sigma \left(W_{2}\sigma \left(W_{1}f_{i}^{t}+b_{1}\right)+b_{2}\right)$$

where $W_{1}$ and $W_{2}$ denote weight matrices, $b_{1}$ and $b_{2}$ represent bias terms, and σ signifies the ReLU activation function.

### 3.3. Relative Spatio-Temporal Coding

**Figure 3:**
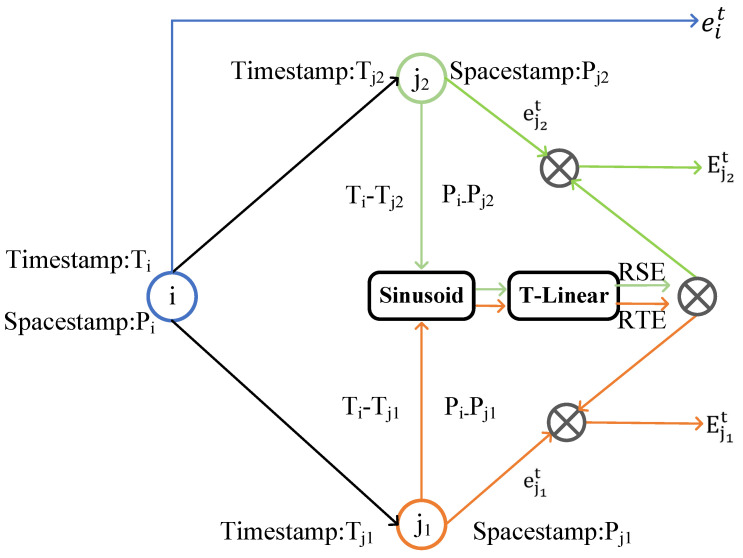
The relative spatio-temporal coding method models the temporal and spatial information of intelligence, with increased representations subsequently input into the temporal Transformer and the multi-spatial GNN following RTE and RSE processing.

The positional encoding mechanism inherent in Transformer models is employed to enhance the modeling of spatio-temporal dependencies in pedestrian trajectory prediction. We propose a relative spatio-temporal encoding strategy derived from this mechanism, which concurrently captures the temporal and spatial relationships among pedestrians, as illustrated in Figure 3. This facilitates interactions among nodes with disparate timestamps and geographic locations, significantly improving the model’s capacity to represent spatio-temporal information.

Specifically, for two pedestrians with different temporal and spatial positions, they are defined as two nodes, *i* and *j*, and their relative time interval can be expressed as $\Delta T_{ij}=T_{i}-T_{j}$, which serves as an index for deriving the relative spatio-temporal coding $RTE_{\Delta Tij}$. Note that the training dataset does not cover all possible time gaps; thus, the relative temporal encoding should be capable of generalizing unseen times and time gaps. Due to the periodicity and smoothness properties of sine and cosine functions, we base our design on the positional encoding mechanism of Transformers and propose a method that combines fixed sinusoidal functions with an adaptable linear projection, denoted as T-Linear*$:R^{d}\to R^{d}$. This combination forms the basis for the relative time encoding of RTE, and the equation is presented as follows:

(3) $$\mathrm{Base}\left(\Delta T\left(i,j\right),2k\right)=\sin\left(\frac{\Delta T(i,j)}{10000^{\left(2k/d\right)}}\right)$$

(4) $$\mathrm{Base}\left(\Delta T\left(i,j\right),2k+1\right)=\cos\left(\frac{\Delta T(i,j)}{10000^{\left(2k+1/d\right)}}\right)$$

(5) $$\mathrm{RTE}\left(\Delta T\left(i,j\right)\right)=\mathrm{T-Linear}\left(\mathrm{Base}(\Delta T(i,j))\right)$$

where *d* represents the dimensionality of the positional encoding, *k* denotes the index of the positional encoding dimension, and Base is the base encoding function that uses sine and cosine functions to encode the time difference ΔT(i,j), capturing spatio-temporal relationships at different frequencies through these encodings.

Similarly, for the source node *i* and the destination node *j*, their spatial coordinates are denoted as $P_{s}=\left(x_{i},y_{i}\right)$ and $P_{n}=\left(x_{j},y_{j}\right)$, respectively. Based on these coordinates, we define the relative spatial encoding (RSE) using the same sinusoidal encoding scheme as in the temporal case, followed by a learnable linear projection. The RSE is computed as

(6) $$\mathrm{RSE}\left(P_{i},P_{j}\right)=\mathrm{T-Linear}\left(\mathrm{Base}(P_{i}-P_{j})\right)$$

Finally, the temporal and spatial encodings are added to the original motion feature vector to form the final representation of the pedestrian node $E_{i}^{t}$, where

(7) $$E_{i}^{t}=e_{i}^{t}+\mathrm{RTE}\left(\Delta T\left(i,j\right)\right)+\mathrm{RSE}\left(P_{i},P_{j}\right)$$

### 3.4. Multiple-Spatio-Temporal-Map Modeling

To thoroughly encapsulate the spatio-temporal interaction data of pedestrians, we propose a methodology for modeling multi-spatio-temporal graphs that amalgamates both temporal and spatial interactions.

We characterize the multimodal spatio-temporal graph using a binary adjacency matrix that delineates the relationship between current observations and historical events as a directed acyclic graph. At time t=2, the directed edges depicted in Figure 1b suggest a possible unilateral interaction between the pedestrians represented by the source node and those identified by the destination node. This interaction may extend throughout a time interval τ={0,…,t−1}, demonstrating how one individual’s historical behavior can affect another’s subsequent choices. Our hypothesis asserts that each pedestrian $x^{t}$ is influenced by their prior behavior $x^{0:t-1}$ as well as by other pedestrians at possibly varying temporal instances.

#### 3.4.1. Spatio-Temporal Graph Construction

Based on the relative spatio-temporal encoding constructed above, the pedestrian node $E_{i}^{t}$ can represent the interactions between pedestrians as a graph structure at any time *t*, where E serves as the node and $R_{ij}^{t}$ denotes the edge. Thus, the graph structure can be expressed as $G_{t}=\left(E_{i}^{t},R_{ij}^{t}\right)$. Ultimately, $G_{t}$ is transformed into the matrix $A_{t}$ and fed into the model for training.

At time *t*, the graph $G_{t}$ is converted into a pedestrian interaction matrix $A_{t}$. Traditional methods rely on the $L_{2}$ norm to measure the distance between pedestrians; however, this approach is susceptible to the influence of distant pedestrians and thus fails to accurately capture the true strength of interactions. To address this limitation, we adopt the inverse of the Euclidean distance as a more effective metric to define the interaction intensity between pedestrians.The following are the formulas for both the $L_{2}$ norm and the inverse of the Euclidean distance:

(8) $$A_{L_{2}}^{ij}=\left\{\begin{array}{cc} {∥V_{t}^{i}-V_{t}^{j}∥}_{2}, & \mathrm{if}\;{∥V_{t}^{i}-V_{t}^{j}∥}_{2}\neq 0\\ 0, & \mathrm{otherwise} \end{array}\right.$$

(9) $$A_{t}^{ij}=\left\{\begin{array}{cc} \frac{1}{{∥V_{t}^{i}-V_{t}^{j}∥}_{2}}, & \mathrm{if}\;{∥V_{t}^{i}-V_{t}^{j}∥}_{2}\neq 0\\ 0, & \mathrm{otherwise} \end{array}\right.$$

where ${∥V_{t}^{i}-V_{t}^{j}∥}_{2}$ denotes the Euclidean distance between two adjacent pedestrians, *i* and *j*, at a specific time *t*. By applying the extension of Equation (9) to the entire scene at time *t*, the interactions among all pedestrians can be systematically assessed in relation to the threshold, thereby facilitating the derivation of the corresponding adjacency matrix of the spatio-temporal map.

#### 3.4.2. Local Judgment

To construct the multi-spatial map, we first encode the pedestrian’s trajectory features using a triplet of feature vectors, analogous to the standard Transformer framework. For pedestrian $p^{i}$ at time *t*, the trajectory information is represented using a query vector *q*, a key vector *k*, and a value vector *v*. These vectors are defined as follows:

(10) $$q_{i}^{t}=f_{q}\left(h_{i}^{t}\right),\;k_{i}^{t}=f_{k}\left(h_{i}^{t}\right),\;v_{i}^{t}=f_{v}\left(h_{i}^{t}\right)$$

where $q_{i}^{t}$ denotes the query vector, $k_{i}^{t}$ denotes the key vector, and $v_{i}^{t}$ signifies the value vector. Additionally, the functions $f_{q}$, $f_{k}$, and $f_{v}$ map to the query, key, and value vectors. Similar to the approach used in self-attention mechanisms, the interaction strength $a_{tt^{\prime }}^{ij}$ between pedestrian $p_{i}$ at time *t* and pedestrian $p_{j}$ at time $t^{\prime }$ is expressed by the following formula:

(11) $$a_{{\mathrm{tt}}^{\prime }}^{ij}=\frac{q_{i}^{t}{\left(k_{j}^{t^{\prime }}\right)}^{T}}{\sqrt{d_{k}}}v_{i}^{t}$$

where $d_{k}$ represents the dimensionality of the vector.

During the local evaluation phase, a dynamic threshold θ is used to determine significant interactions among pedestrians. The threshold θ is adaptively adjusted based on the mean and standard deviation of interaction influences across all pedestrians, as shown below:

(12) θ=μ+w·σ

where μ denotes the mean interaction influence, σ is the standard deviation, and *w* is a coefficient controlling the threshold’s sensitivity.

Extending Equation (11) to the entire scenario, interactions between all pedestrians at time $t^{\prime }$ and pedestrian *i* at time *t* are represented in matrix form as $A_{t}^{ij}$. The element $a_{tt^{\prime }}^{ij}$ denotes the interaction strength between pedestrian *i* at time *t* and pedestrian *j* at time $t^{\prime }$.

Through the local judgment formula, the interaction strength between any two pedestrians at different spatio-temporal points can be evaluated, enabling the determination of interactions across varying spatio-temporal contexts. The local judgment formula is given as follows:

(13) $$A_{tt^{\prime }}^{ij}=\left\{\begin{array}{cc} 1, & \mathrm{if}\;a_{tt^{\prime }}^{ij}>\theta \\ 0, & \mathrm{otherwise} \end{array}\right.$$

#### 3.4.3. Global Judgment

**Figure 4:**
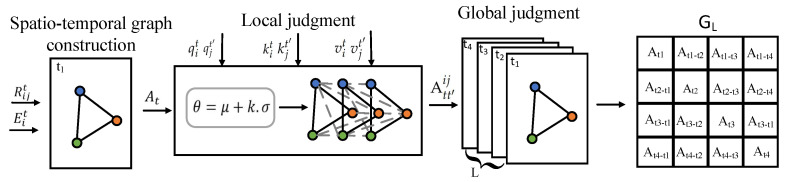
In multiple-spatio-temporal mapping, various spatio-temporal maps are generated by layering maps from distinct time points. This entails computing the weights of pedestrians at different intervals concerning the current target pedestrians, conducting a localized assessment to discern any influences, and selecting L moments with the most significant interactions with the present moment following a global evaluation to execute the multiple-spatio-temporal-map modelling.

During the global judgement phase, we assess the cumulative impact of all pedestrians at time *t* on pedestrian $p^{i}$ at time $t^{\prime }$:

(14) $${\mathrm{Impact}}_{i}^{t}=\sum_{j=1}^{N}A_{{\mathrm{tt}}^{\prime }}^{ij}$$

where *N* denotes the total number of pedestrians at time $t^{\prime }$, and $A_{tt^{\prime }}^{ij}$ represents the influence of pedestrian $p^{i}$ at time *t* on pedestrian $p^{j}$ at time $t^{\prime }$. The time instances are scored based on their influence, and the top *L* moments with the highest impact are selected. Subsequently, the graphs corresponding to these *L* moments are superimposed to form a multi-spatial graph consisting of *L* layers. This process yields an augmented adjacency matrix $G_{L}$, as illustrated in Figure 4.

### 3.5. Neural Networks for Multi-Spatial Graphs

The primary function of this module is to integrate information between previously connected nodes through the use of graph attention networks (GATs), continuously updating the node features. It can be conceptualized as a message-passing architecture within an undirected graph. These networks operate by calculating attention weights for each node relative to its neighbors, thereby enabling the extraction of additional information from the overall structure of the graph.

**Figure 5:**
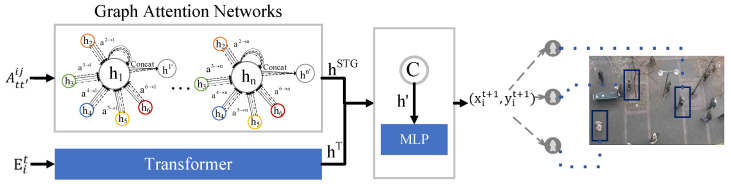
We employ a GAT and a Transformer network to jointly capture pedestrian interaction dynamics. The GAT processes spatio-temporal interactions among pedestrians to generate $h^{STG}$, while the Transformer focuses on learning long-term interactions and produces $h^{T}$. Finally, $h^{STG}$ and $h^{T}$ are concatenated and fed into the decoder to generate real-world 2D coordinates.

The multi-spatial graph neural network consists of multiple graph attention layers. Each graph attention layer processes the node features $E_{i}^{t}$ and the augmented adjacency matrix $G_{L}$ as inputs, generating the expected trajectory features shown in Figure 5. The model employs multi-head attention, characterized by four distinct heads represented by dashed, solid, dotted, and dash-dot lines. Upon obtaining various node features, each head consolidates these features to derive the final trajectory feature $h^{STG}$.

Concurrently, to examine the temporal dependencies of pedestrians and variations in their intentions, the trajectory attributes of an individual pedestrian are recorded over time using a separate Transformer. This Transformer sequentially inputs the trajectory features $E_{i}^{t}$ to generate future trajectory features $h^{T}$. Subsequently, $h^{STG}$ is combined with $h^{T}$ to yield the final feature $h^{\prime }$, which is then fed into the MLP decoder to be translated into real-world trajectory coordinates.

However, as the complexity of the model increases, particularly with the application of multi-layer graph attention mechanisms, the potential problem of overfitting arises. To address this, the present study employs the dropout method, where 10% of the neurons are randomly dropped in each layer of the neural network, thereby enhancing the model’s generalization capability. Additionally, early stopping is applied during training to ensure that the model ceases training once the performance on the validation set stops improving.

## 4. Experiments

### 4.1. Datasets and Metrics

To assess the proposed algorithms and models, we performed experimental validation on four datasets and conducted comprehensive analyses of the results. These datasets include ETH/UCY [23,23], SDD [24], and the SportVU NBA sports dataset which focused on the NBA game data from the 2015–2016 season. Given the large size of the original dataset, one of its sub-datasets named “Rebounding” was selected for benchmark testing, containing 257,230 twenty-frame trajectories. We then executed simulation experiments and ablation studies using the leave-one-out cross-validation protocol [41] to verify efficacy and deepen our insights. The ETH and UCY capture densely populated settings such as hotels and streets. The SDD dataset supplies pedestrian and vehicle trajectories from complex overhead views. Finally, the SportVU NBA dataset provides detailed movement trajectories of NBA basketball players, featuring extensive player interactions and strategic movements, which are used to rigorously evaluate model performance in a highly dynamic and complex sports environment.

We selected STGformer as the baseline model for our overall performance evaluation, allowing for a comprehensive comparison with state-of-the-art spatio-temporal graph Transformer methods. Since MSTT is built upon the STAR architecture through the addition of new modules, we chose the original STAR model as the baseline for our ablation studies. In this setting, we incrementally added each proposed module to STAR to quantify its individual contribution to the overall performance.

To assess the precision of the trajectories forecasted by the different components and the model, we utilize the following metrics: the average displacement error (ADE) and the final displacement error (FDE). The ADE is characterized as the mean Euclidean distance between the actual position at each predicted point in the trajectory, represented as $\hat{x_{i}^{t}}$, and the projected value $x_{i}^{t}$. The formula for the ADE is expressed as follows:

(15) $$\sigma_{\mathrm{ADE}}=\frac{1}{N}\sum_{i=1}^{N}\frac{1}{T}\sum_{t=1}^{T}\left|\hat{x_{i}^{t}}-x_{i}^{t}\right|$$

Similarly, the FDE represents the Euclidean distance between the actual endpoint location $\hat{x_{i}^{T}}$ and the estimated endpoint location $x_{i}^{T}$, articulated as

(16) $$\sigma_{\mathrm{FDE}}=\frac{1}{N}\sum_{i=1}^{N}\left|\hat{x_{i}^{T}}-x_{i}^{T}\right|$$

### 4.2. Experimental Details

To ensure comparability with prior work, such as the classical methods Social-GAN [14], STAR [16], and Trajectron++ [10], while maintaining sufficient trajectory information and avoiding the introduction of redundancy, this study employs the first 8 frames as the observation sequence and the subsequent 12 frames as the prediction target sequence. Training utilizes historical data from four datasets, while the fifth dataset is reserved as the test set for evaluating the model’s correctness. This procedure is reiterated to guarantee that each dataset functions as the test set a single time. To ensure equity, all baseline models adhere to an identical training protocol and are assessed on an Nvidia GTX4050Ti GPU.

The model utilizes the Adam optimizer with a batch size of 16 and a learning rate of 0.0015, and it is trained for 300 epochs, with each batch comprising around 256 pedestrian trajectory data points from various time frames. The threshold *w* in Equation (12) is set to 0.5 m, a value determined through ablation experiments to optimally balance adjacency sensitivity and robustness. The number of layers *L* in the multi-relational spatio-temporal graph module is fixed at 5, based on a trade-off analysis between performance and computational cost. All Transformer layers employ an input feature dimension of 32. Furthermore, ablation studies on the attention head count in both GAT and Transformer modules demonstrate that utilizing four attention heads achieves optimal ADE/FDE metrics, consequently improving overall model performance.

### 4.3. Quantitative Evaluation

Table 1 presents a comparative analysis between the proposed model and existing models across benchmark datasets. Our model demonstrates superior performance in terms of both ADE and FDE metrics, attributable to its multi-relational spatio-temporal network architecture and relative spatio-temporal encoding scheme. These mechanisms effectively capture dynamic pedestrian interactions, yielding a more precise representation of inter-agent influences. On the ZARA2 dataset, our model achieves the optimal results. While ranking second in terms of the ADE and FDE compared to STGformer, it exhibits a more balanced overall performance.

We compare the proposed model with state-of-the-art baselines on the SDD and SportVU NBA datasets. As shown in Table 2, our model achieves the best results on both datasets. On SDD, we obtain an ADE of 3.16 and an FDE of 5.12, representing 41.3% and 42.6% improvements over STGformer [41], respectively. For the Rebounding subset of the NBA data, our method achieves an ADE/FDE of 11.36/13.42, outperforming STGformer by 8.5% and 13.4%, respectively. These results demonstrate our model’s superior capability in capturing complex spatio-temporal interactions across both pedestrian and sports movement scenarios.

In practical applications, particularly within the field of autonomous driving where the accurate prediction of pedestrian behavior is of utmost significance, the inference time of a model is critically important. To assess the effectiveness of the proposed model, a comparison is conducted with the PECNET, STGformer, STAR, and SocialCircle+ models based on the number of parameters and inference time. The proposed model exhibits an increase of $3.8\times 10^{3}$ parameters relative to PECNET while achieving a reduction of 0.006 s in inference time. When compared to STGformer, the suggested model contains $2.0\times 10^{3}$ fewer parameters and achieves a decrease in inference time by 0.015 s. In relation to the STAR model, the proposed model integrates an additional $8.3\times 10^{3}$ parameters, resulting in a 0.015 s increase in inference duration. Lastly, in comparison to the STGformer model, the proposed model features $5.6\times 10^{3}$ fewer parameters and demonstrates a reduction in inference time of 0.028 s.

Table 3 presents a comparison of five trajectory prediction models on the ETH/UCY dataset in terms of the parameter count, memory usage, and per-trajectory inference latency. Among them, STGformer has the largest number of parameters, corresponding to the highest inference latency, while STAR has the smallest parameter count and achieves the lowest latency. In contrast, the proposed method maintains a relatively low inference latency (0.158) with moderate memory usage (13.5), demonstrating a favorable balance between computational efficiency and resource consumption.

**Figure 6:**
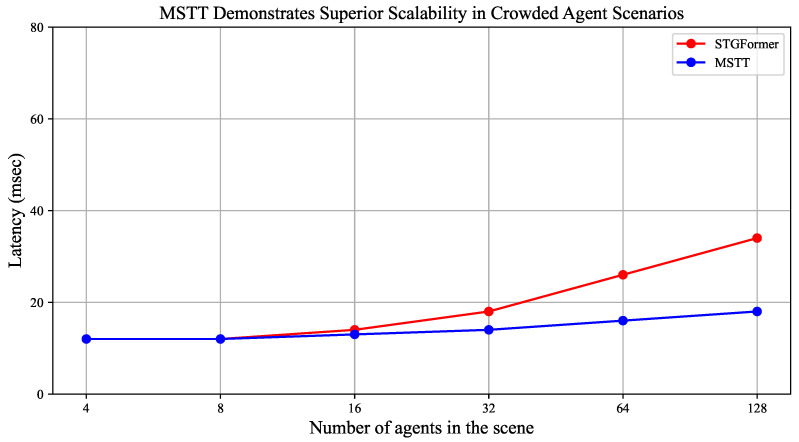
The inference latency of two models (MSTT and STGformer). The red curve represents the STGformer model, while the blue curve represents the MSTT model.

Figure 6 presents a comparison of inference latency between the MSTT and STGformer models under varying numbers of agents. As the number of agents increases from 4 to 128, MSTT exhibits a modest latency increase from 12 ms to 18 ms, while the latency of STGformer rises sharply from 12 ms to 34 ms. This trend demonstrates the superior scalability and stability of MSTT in densely populated scenarios.

### 4.4. Qualitative Analysis

**Figure 7:**
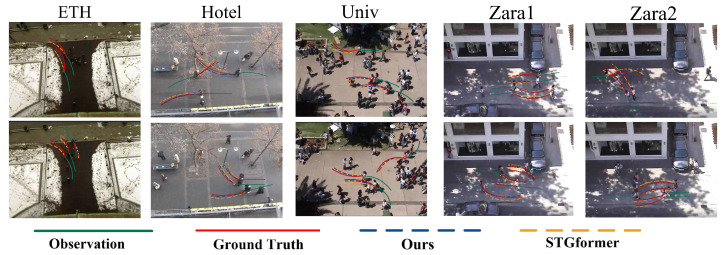
Qualitative results for the ETH/UCY dataset. For each pedestrian, we show the path history (green line), the future true path (red line), the STGformer prediction results (yellow dashed line), and the prediction results of our MSTT (blue dashed line).

Pedestrian mobility involves individual interactions that result in intricate behaviors such as following, collision evasion, and navigation. Therefore, precise modeling of these interactions is essential. We conducted a thorough investigation of the MSTT model’s predictive efficacy across various motion patterns and collision avoidance scenarios. Ten example scenarios were selected to assess the MSTT model’s performance compared to the STGformer model, particularly in complex pedestrian interaction scenarios, as shown in Figure 7.

The experimental results demonstrate that both the MSTT and STGformer models efficiently capture pedestrian interactions and generate comparable trajectories in most cases. However, the trajectories forecasted by the MSTT model align more closely with observed behaviors. The MSTT model employs a multi-spatial modeling strategy that thoroughly analyzes interactions among diverse pedestrians and explores social-temporal relationships in more detail, yielding trajectories that are more coherent and fluid than those generated by the STGformer model.

**Figure 8:**
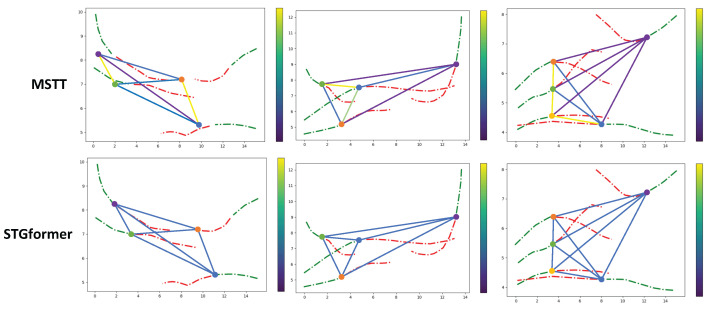
Graph representation of pedestrian interactions. Nodes denote individual pedestrians. Edge colors ranging from purple to yellow correspond to interaction weights computed by the GAT, with the color bar indicating the weight scale. Green dashed lines display historical trajectories across 8 frames while red dashed lines show ground truth trajectories spanning 12 frames.

Figure 8 shows the connection between weight visualizations in the two methodologies. Notably, the MSTT model generally allocates higher weights to pedestrians located both temporally and spatially closer. Furthermore, the weight values in the MSTT model display more variability compared to STGformer. This variability arises from the MSTT model’s ability for relative spatio-temporal coding and multiple-spatio-temporal modeling, which allows it to recognize important interactions with pedestrians more accurately, resulting in varied weight assignments.

**Figure 9:**
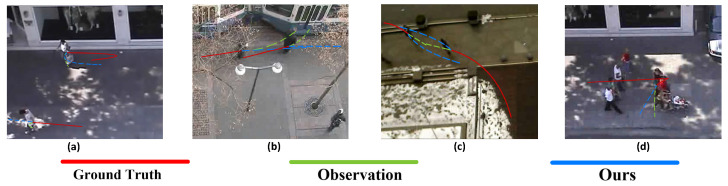
Qualitative evaluation reveals four characteristic failure modes: (**a**) peripheral observation artifacts, (**b**) occlusion-induced deviations, (**c**) collective motion discontinuities, and (**d**) high-curvature path miscalculations, where red, green and blue trajectories denote ground truth, observed data, and model predictions respectively, and the prediction results of our MSTT (blue dashed line).

To analyze the limitations of MSTT, we perform a qualitative error analysis by examining representative failure cases and visualizing them in Figure 9. In Figure 9a,b, when pedestrians approach the boundary of the scene, where insufficient historical trajectory and environmental context hinders intent inference, the model produces significantly deviated predictions. In Figure 9c,d, sudden, nonlinear changes in direction or speed similarly degrade the predictive accuracy of the model.

### 4.5. Ablation Experiments

To evaluate the effectiveness of the relative spatio-temporal encoding (RSTE) and multi-spatio-temporal graph modeling (MSTG), we design incremental ablation experiments based on the STAR model, gradually integrating each component to compare performance. The original STAR model is first used as a baseline, without incorporating any complex encoding or graph modeling. Then, two models are constructed: STAR-R, which includes only RSTE, and STAR-M, which includes only MSTG. All models are tested using the same number of samples. As shown in Table 4, RSTE reduces the ADE and FDE by 0.04 and 0.08, respectively, while MSTG reduces them by 0.03 and 0.10, indicating that both components significantly contribute to performance improvement.

To evaluate the effectiveness of the proposed strategy, a comprehensive set of ablation experiments was conducted to examine the performance of each submodule within the ETH and UCY datasets. This was achieved while maintaining uniform configurations for the remaining modules in relation to the final model. The experimental results are presented in Table 4, where the underlined elements indicate the combinations implemented in the final model. The removal of any component leads to a decline in the efficacy of pedestrian trajectory prediction.

We demonstrate that parameter configurations exert a substantial influence on prediction accuracy. As presented in Table 5, increasing the number of attention heads from 1 to 4 reduces the average ADE/FDE from 0.24/0.35 to 0.20/0.31, representing a marked improvement. However, increasing the number of heads to eight reduces performance to 0.22/0.37, indicating that four heads achieve the optimal balance between representational capacity and generalization. For the hyperparameter *w*, the highest accuracy of 0.20/0.31 is achieved when w=0.5, while values of 0.25 or 0.75 lower performance to 0.24/0.36 and 0.22/0.33, suggesting that a moderate weighting coefficient better balances local and global relational information. In terms of distance metrics, constructing the adjacency matrix using the inverse of the Euclidean distance yields a performance of 0.20/0.31, surpassing the $L_{2}$-norm method with 0.22/0.33, confirming that the inverse of the Euclidean distance more effectively captures the interaction intensity among pedestrians. These parameter refinements consistently improve performance across all five evaluation scenarios.

### 4.6. Optimal Graph Stacking Number Analysis

To evaluate the effect of the spatial layer count L in the multi-space module described in the Section 3.4.3, we conducted an ablation study by varying the number of spatial layers. This parametric analysis aimed to identify the optimal hierarchical configuration that balances predictive performance with computational efficiency. Specifically, while keeping all other parameters fixed, we varied only the number of spatial layers L and recorded two key metrics $\sigma_{ADE}$ and $\sigma_{FDE}$ for each value of L. At the same time, we monitored the corresponding computational resource consumption to determine the most effective graph stacking depth.

**Figure 10:**
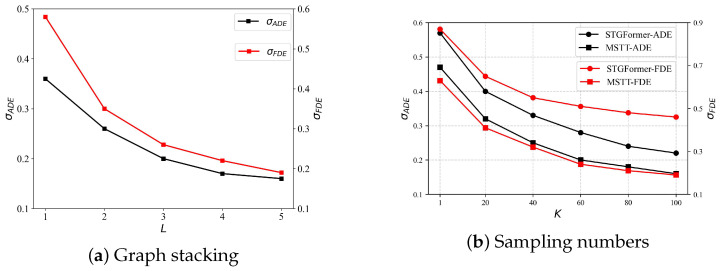
(**a**) Analysis of various graph stacking numbers and their effect on the MSTT model’s efficacy; (**b**) evaluation of differing sampling numbers in relation to ADEs (black line) and FDEs (red line) for two models.

As illustrated in Figure 10a, both the $\sigma_{ADE}$ and $\sigma_{FDE}$ metrics exhibit a consistent decreasing trend with increasing layer counts. It is essential to recognize that an increase in *L* leads to a quadratic expansion of the adjacency matrix, substantially increasing the time and processing resources required for training and testing. Our data indicates that while the error continuously diminishes, the rate of that diminution reduces with the addition of more layers. This indicates that the performance improvements from incorporating additional spatial layers do not warrant the increased processing requirements. Therefore, to achieve a compromise between the computing economy and performance enhancement, we determine that five spatial layers are optimal for this investigation, offering a significant performance increase without imposing excessive processing demands.

To assess the model’s ability to generalize, the sample size was systematically increased, with the experimental outcomes presented in Figure 10b, which shows that an increase in the parameter *K* correlates with a decrease in both the ADE and FDE metrics. Moreover, for an equivalent sample size, the proposed model demonstrates superior performance compared to the STGformer model, indicating that it requires fewer samples to achieve a similar error rate. These results suggest that integrating the multi-relational spatio-temporal module effectively captures pedestrian interactions across various spatio-temporal contexts, reducing variance in predicted trajectories and improving both prediction accuracy and generalization performance.

## 5. Summary

We propose a pedestrian trajectory prediction model based on multi-relational spatio-temporal graphs (MSTT), which addresses the shortcomings of traditional approaches in capturing dynamic and complex human interactions. Conventional methods often rely on static interaction structures, rendering them insufficient for modeling evolving spatio-temporal dependencies. To overcome this, MSTT integrates relative spatio-temporal encoding with multi-relational graph modeling, thereby enhancing the model’s capacity to represent dynamic inter-agent behaviors. Experimental results show that MSTT achieves substantial accuracy improvements on the SDD and NBA datasets. Although there remains room for further improvement on the ETH and UCY datasets, MSTT exhibits clear advantages over STGformer in terms of inference efficiency, scalability, and the parameter economy. Comparative and ablation studies further validate the effectiveness and balanced design of the proposed model.

## Figures and Tables

**Table 1:** Performance comparison (ADE/FDE in meters) across methods. Bold values indicate the best-performing model on each dataset. The model uses 8 observed time steps to predict the next 12.

Model	Year	ETH	HOTEL	UNIV	ZARA1	ZARA2	Avg
Social-GAN [14]	2018	0.73/1.48	0.49/1.01	0.41/0.84	0.27/0.56	0.33/0.70	0.45/0.91
PECNet [27]	2019	0.81/1.52	0.72/1.61	0.60/1.26	0.34/0.69	0.42/0.84	0.58/1.18
Trajectron++ [10]	2020	0.67/1.18	0.18/0.28	0.30/0.54	0.25/0.41	0.18/0.32	0.32/0.55
STAR [16]	2020	0.36/0.65	0.21/0.36	0.31/0.62	0.26/0.55	0.22/0.46	0.27/0.53
DERGCN [18]	2023	0.54/1.01	0.23/0.42	0.30/0.63	0.22/0.44	0.20/0.42	0.30/0.58
PPT [16]	2024	0.35/0.51	0.15/0.25	**0.13**/0.24	0.22/0.39	0.18/0.31	0.21/0.34
STIGCN [40]	2024	0.42/0.58	0.14/0.23	0.17/0.29	0.26/0.45	0.21/0.37	0.24/0.38
SocialCircle+ [42]	2024	0.25/**0.42**	**0.10/0.15**	0.24/0.42	0.23/0.38	0.18/0.24	0.20/0.32
STGformer [41]	2024	0.27/0.56	0.11/0.17	0.18/**0.23**	**0.16/0.30**	0.17/0.21	**0.18/0.29**
MSTT(Ours)	-	**0.24**/0.49	0.18/0.29	0.19/0.28	0.21/**0.30**	**0.16**/**0.19**	0.20/0.31

**Table 2:** Results of trajectory prediction for SDD and Rebounding using ADE/FDE metrics in pixels comparing our proposed method (MSTT) with related methods from the literature. The lower error indicates better performance, and the best performance is marked in bold.

Dataset	Social-GAN [14]	STAR [16]	DERGCN [18]	STGformer [41]	Ours
SDD	27.23/41.44	7.85/11.85	8.21/10.22	5.38/8.92	**3.16**/**5.12**
Rebounding	30.54/47.68	15.65/19.21	14.06/17.63	12.42/15.49	**11.36**/**13.42**

**Table 3:** Analysis of model inference duration and parameter count. The inference time denotes the duration required for one trajectory in the UCY dataset.

Model Name	Parameter Count (M)	Memory Usage (GB)	Inference Latency (ms)
PECNet [27]	25.0	10.5	0.164
SocialCircle+ [42]	30.8	12.6	0.173
STAR [16]	20.5	14.4	0.143
STGformer [41]	34.4	16.4	0.186
Ours	28.8	13.5	0.158

**Table 4:** Ablation experiments for the model. Each entry contains two numerical values representing the ADE/FDE of the predicted outcomes. Observations are recorded for 8 time steps, while the predicted values pertain to the subsequent 12 time steps.

Model	ETH	HOTEL	ZARA1	ZARA2	UNIV	Avg
STAR	0.36/0.65	0.22/0.36	0.27/0.56	0.32/0.55	0.28/0.68	0.27/0.53
STAR-R	0.33/0.52	0.22/0.34	0.25/0.50	0.22/0.45	0.24/0.58	0.25/0.48
STAR-M	0.34/0.58	0.18/0.28	0.24/0.41	0.25/0.48	0.27/0.54	0.26/0.46
STAR-R-M	0.24/0.49	0.18/0.29	0.19/0.28	0.21/0.30	0.16/0.19	0.20/0.31

**Table 5:** Accuracy comparison across experimental variants. Values underlined indicate optimal component configurations and bold values mark overall best performance. The evaluation protocol uses 8 observed frames to predict subsequent 12-frame trajectories.

Method	Variants	ADE/FDE					
		**ETH**	**HOTEL**	**UNIV**	**ZARA1**	**ZARA2**	**Avg**
w	0	0.42/0.67	0.32/0.45	0.31/0.39	0.28/0.48	0.26/0.24	0.32/0.45
	0.25	0.32/0.54	0.26/0.30	0.21/0.36	0.22/0.35	0.18/0.23	0.24/0.36
	0.5	0.24/0.49	0.18/0.29	0.19/0.28	0.21/0.30	0.16/0.19	**0.20/0.31**
	0.75	0.28/0.42	0.21/0.32	0.23/0.33	0.23/0.35	0.17/0.22	0.22/0.33
Multi-head	w/o	0.38/0.65	0.28/0.42	0.30/0.45	0.32/0.40	0.26/0.34	0.31/0.45
	1	0.29/0.54	0.21/0.26	0.23/0.45	0.24/0.26	0.21/0.26	0.24/0.35
	2	0.26/0.51	0.18/0.26	0.20/0.46	0.20/0.32	0.17/0.23	0.20/0.36
	4	0.24/0.49	0.18/0.29	0.19/0.28	0.21/0.30	0.16/0.19	**0.20/0.31**
	8	0.27/0.52	0.20/0.26	0.19/0.48	0.25/0.34	0.18/0.25	0.22/0.37
WeightA	w/o	0.30/0.56	0.25/0.36	0.24/0.32	0.26/0.33	0.18/0.23	0.25/0.36
	$A_{L2}$	0.26/0.53	0.20/0.30	0.20/0.31	0.24/0.30	0.18/0.22	0.22/0.33
	$A_{t}$	0.24/0.49	0.18/0.29	0.19/0.28	0.21/0.30	0.16/0.19	**0.20/0.31**

## Data Availability

The original contributions presented in this study are included in the article. Further inquiries can be directed to the corresponding author.
